# Pituitary xanthogranulomas: clinical features, radiological appearances and post-operative outcomes

**DOI:** 10.1007/s11102-017-0859-x

**Published:** 2018-01-23

**Authors:** R. Ved, N. Logier, P. Leach, J. S. Davies, C. Hayhurst

**Affiliations:** 1 B4 Office, Department of Neurosurgery, University Hospital of Wales, Cardiff, CF 14 4XW UK; 2 Department of Endocrinology, University Hospital of Wales, Cardiff, CF 14 4XW UK

**Keywords:** Xanthogranuloma, Pituitary, Sellar, Cystic

## Abstract

**Background:**

Xanthogranulomas are inflammatory masses most commonly found at peripheral sites such as the skin. Sellar and parasellar xanthogranulomas are rare and present a diagnostic challenge as they are difficult to differentiate from other sellar lesions such as craniopharyngiomas and Rathke’s cleft cysts pre-operatively. Their radiological imaging features are yet to be clearly defined, and clinical outcomes after surgery are also uncertain. This study reviews clinical presentation, radiological appearances, and clinical outcomes in a cohort of patients with pituitary xanthogranulomas.

**Methods:**

A prospectively maintained pituitary surgery database was screened for histologically confirmed pituitary xanthogranulomas between May 2011–December 2016. Retrospective case note assessments were then performed by three independent reviewers. Patient demographics, clinical presentations, imaging, and clinical outcomes were analysed.

**Results:**

During the study period 295 endoscopic endonasal pituitary surgeries were performed. Six patients had confirmed pituitary xanthogranulomas (2%). Patients most commonly presented with visual field deficits and/or endocrine dysfunction. Common imaging features included: a cystic consistency, hyperintensity on T1-weighted MR images, and contrast enhancement either peripherally (n = 3) or homogenously (n = 3). The most common pre-operative endocrine deficits were hyperprolactinaemia and hypoadrenalism (at least one of which was identified in 4/6 patients; 66%). Thirty-three percent (2/6) of patients presented with diabetes insipidus. The most common post-operative endocrinological deficits were adrenocortical dysfunction (66%) and gonadotropin deficiency (66%). Visual assessments normalised in all six patients post-operatively. Gross total resection was achieved in all patients, and at median follow up of 33.5 months there were no cases of tumour recurrence.

**Conclusions:**

The prevalence of pituitary xanthogranulomas in our series is higher than that suggested in the literature. Surgery restored normal vision to all cases, however four patients (67%) required long-term hormonal replacement post-operatively. Imaging features such peripheral rim enhancement, a suprasellar tumour epicentre, and the absence of both calcification or cavernous sinus invasion were identified as potential indicators that together should alert clinicians to the possibility of pituitary xanthogranuloma when assessing patients with cystic sellar and parasellar tumours.

## Introduction and aims

Xanthogranulomas are a pathological entity thought to result from a process of chronic inflammation. Characteristic histology findings include foamy macrophage infiltration, central necrosis, multinucleated giant cells and haemosiderin deposition [1]. They most commonly develop at peripheral sites, such as the skin or eye. They rarely develop intracranially, and only a handful of case reports and series describe xanthogranulomas developing in the parasellar or sellar regions, with their prevalence amongst pituitary tumour patients reported at ~ 0.6% [2]. They are often only diagnosed post-operatively, due to their clinical and radiological similarity to more common sellar lesions such as craniopharyngiomas (CPs) and Rathke’s cleft cysts (RCCs). Due to the rarity of xanthogranulomas, the long-term endocrine and clinical outcomes have been largely unexplored. Similarly defining radiological appearances to aid pre-operative diagnosis and operative planning have not been well described.

This retrospective study reviews the clinical presentation, radiological appearances, endocrine outcomes, and tumour recurrence rates in a cohort of patients with confirmed pituitary xanthogranulomas.

## Methods

**Fig. 1:**
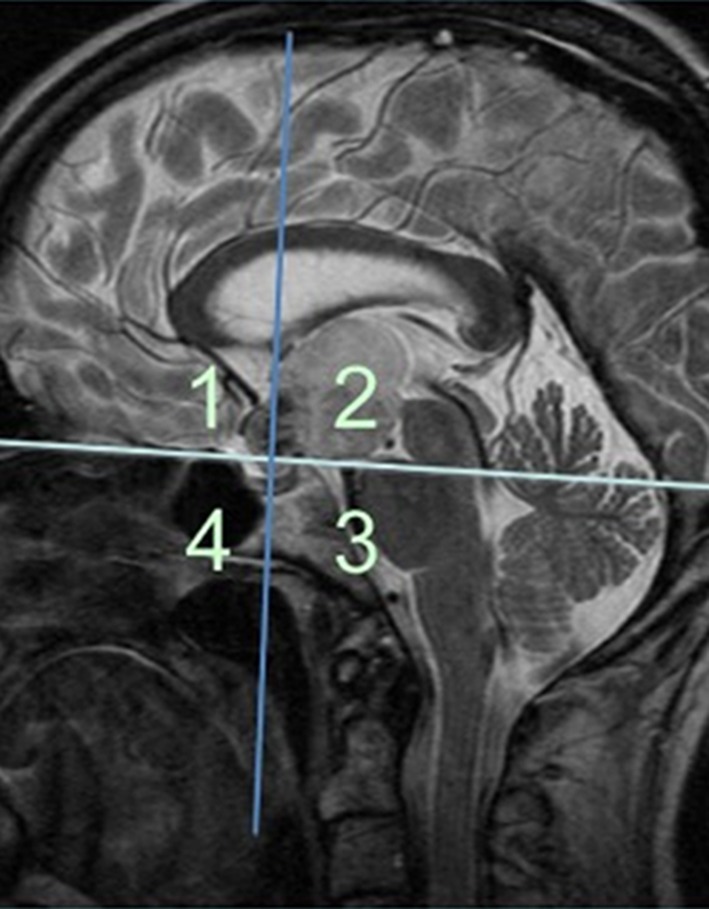
The method utilised to evaluate suprasellar tumour growth, adapted from Petrakakis et al. The four quadrants used to define tumour expansion are outlined; the quadrant housing the tumour epicentre, and growth within each quadrant, can be assessed individually. Quadrant 1 represents the suprasellar preinfundibular region, quadrant 2 the suprasellar retroinfundibular region, quadrant 3 the clival sellar region, and quadrant 4 the nasal sellar region. Adapted from: Petrakakis et al. [3] The sellar and suprasellar region: A “hideaway” of rare lesions. Clinical aspects, imaging findings, surgical outcome and comparative analysis. Clinical Neurology and Neurosurgery. 149:154–165

Histologically confirmed cases of pituitary xanthogranuloma between May 2011–December 2016 were identified from a prospectively maintained pituitary surgery database at a single neurosurgical unit. A retrospective case note review was performed by three independent reviewers to collect data on patient demographics, presenting symptoms, endocrine function pre-and post-operatively, visual outcomes and tumour recurrence. Pre-operative MR imaging was reviewed by a senior neurosurgeon. Maximum tumour diameter, tumour consistency, (solid/cystic) enhancement pattern, T1/T2 signal characteristics and cavernous sinus involvement were recorded. The method of evaluating parasellar tumour growth pattern and origin introduced by Petrakakis et al. [3] was utilised. This involves division of the parasellar region into 4 quadrants on a midline sagittal T2-weighted (T2W) image to evaluate the growth pattern of pituitary lesions (Fig. 1). The 4 quadrants are defined by two hypothetical lines, one connecting the planum sphenoidale and the dorsum sellae, (light blue line) and another, (dark blue) crossing perpendicularly to the first line where the pituitary stalk would descend through the diaphragma sellae. This paradigm permits comparison of the tumour epicentres, and can help define differential growth patterns of sellar and parasellar tumours.

## Results

### Demographics and presenting features

During the study period 295 patients underwent endoscopic endonasal pituitary surgery, of which 6 (2%) had histologically confirmed xanthogranulomas. Four patients (67%) were female and 2 were male. The mean age of patients at presentation was 28.6 years (range 10–63 years). The mean duration of symptoms before a diagnosis made was 6.6 months (median 5 months, range 1–18 months).

The most common presenting symptoms were visual deterioration (4/6; 67%), endocrinological dysfunction (4/6; 67%), and headaches (3/6; 50%). Patients presented with a range of endocrine issues, (Table 1). Two patients (33%) presented with polyuria and polydipsia, one of which met the medical criteria for diabetes insipidus. Only one patient (17%) presented with irregular periods and galactorrhoea, however biochemical hyperprolactinaemia was subsequently confirmed in a total of 4 cases (67%) within the cohort. Pre-operative trials of a dopamine agonists (bromocriptine or carbergoline) in three of these patients had no influence upon on tumour size or prolactin levels. Biochemical adrenal and thyroid axis deficiency was identified in 4 (67%) patients at presentation. Gonadal axis dysfunction was noted in two patients (33%). One patient (17%) had biochemical growth hormone deficiency pre-operatively.

**Table 1:** Patient demographics and presenting features in the pituitary xanthogranuloma patient cohort

Case	1	2	3	4	5	6
Gender	Female	Female	Female	Male	Male	Female
Age at presentation	63	39	17	25	18	10
Presenting symptoms	Polyuria and polydipsia	Irregular periods, galactorrhoea	History of visual deterioration	Headaches in frontal parietal region and visual defects	Sudden onset of headache, polyuria and polydipsia, visual defects	Intermittent headaches and deterioration of left eye vision
Pituitary function at diagnosis						
Corticotropin axis	–	–	Normal	–	–	Normal
Thyrotropin axis	–	–	Normal	–	–	Normal
Gonadotropin axis	Normal	–	Normal	Normal	–	Normal
Somatrotropin axis	Normal	–	Normal	Normal	Normal	Normal
ADH axis	–	Normal	Normal	Normal	Normal	Normal
Prolactin	+ (1364)	+ (2823)	+ (1881)	+ (462)	Normal	Normal
Pre-operative treatment	Bromocriptine trial, hydrocortisone, thyroxine and desmopressin	Cabergoline trial	Cabergoline trial	Hydrocortisone and levothyroxine	None	None
Duration of symptoms before diagnosis	3 months	8 months	7 months	3 months	1 month	18 months
Co-morbidities	Smoker	Obesity	None	None	Obesity	None

– indicates deficient, + indicates excess

All patients underwent surgery, either transsphenoidal hypophysectomy, (5/6) or craniotomy (1/6) via techniques described previously [4]. The pituitary stalk was preserved in all but one case, wherein adherence of the tumour to the stalk necessitated sacrifice of the structure to obtain adequate tumour clearance as the pre-operative radiological diagnosis was thought to be craniopharyngioma. Intra-operative frozen section analysis was not undertaken in this case. Gross total resection (GTR) was achieved in all cases, defined as the absence abnormal enhancing tissue on post-op imaging performed within 8 weeks of surgery.

### Pre-operative imaging features

**Fig. 2:**
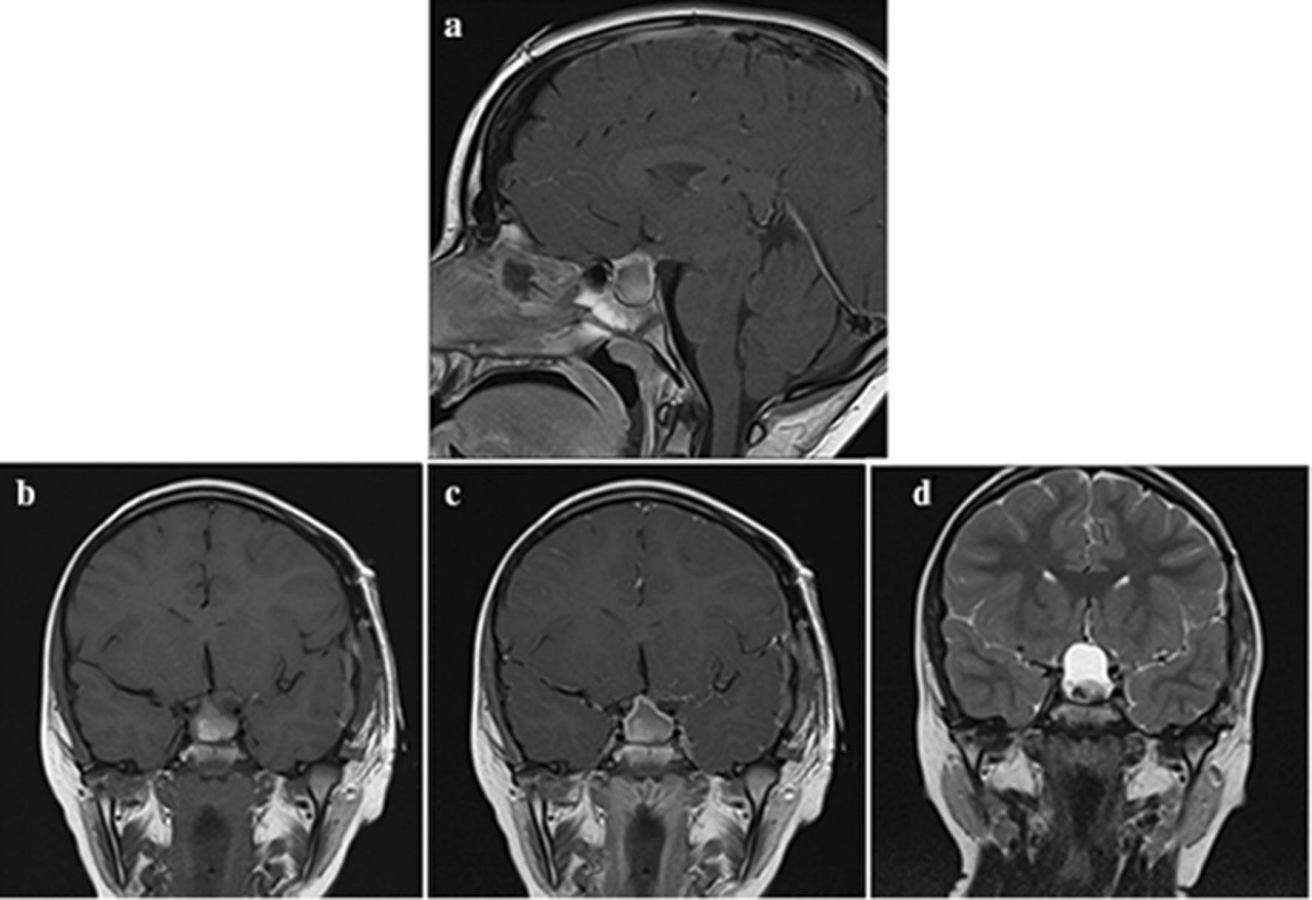
Case 5—Exemplar MR imaging findings in pituitary xanthogranuloma. There is a cystic lesion demonstrating peripheral rim contrast enhancement, and a tumour epicentre identifiable in quadrant 1. **a** T1 weighted sagittal (post-contrast); mixed intensity lesion with predominantly high T1W signal centrally, and peripheral rim enhancement. **b** T1 weighted coronal (pre-contrast); mixed intensity lesion with predominantly high T1W signal centrally. **c** T1 weighted coronal (post-contrast); mixed intensity lesion with predominantly high T1W signal centrally. The peripheral rim enhancement is easily appreciated in this image. **d** T2 weighted coronal; mixed intensities within the lesion, suggestive of the presence of both solid and cystic components

**Fig. 3:**
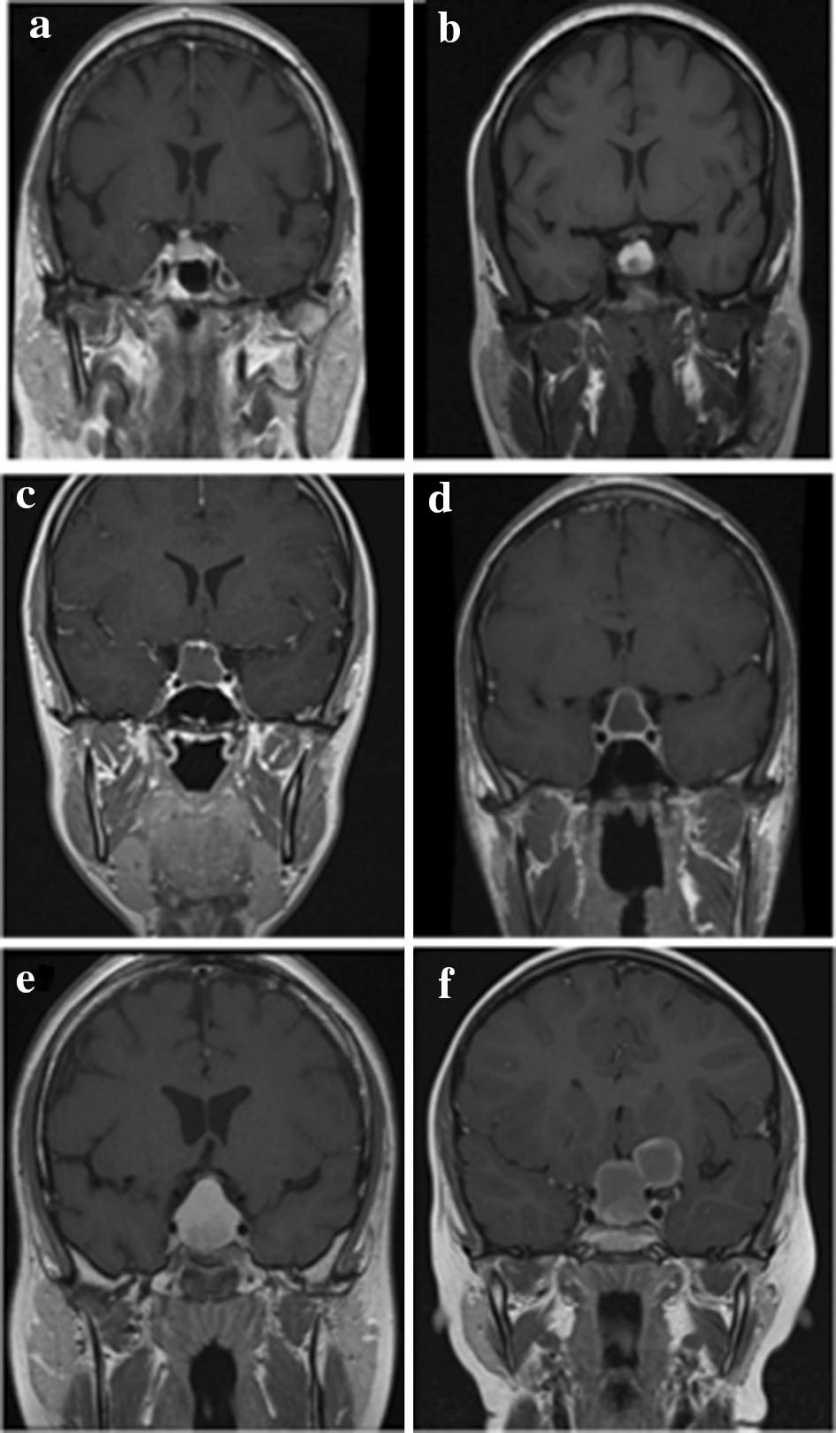
**a** Representative T1W MR image of case (1) the T1W hyperintense sellar lesion is noted. **b** Representative T1W MR image of case (2) the lesion exhibits T1W hyperintensity, with a degree of heterogeneous signal centrally. **c** Representative T1W MR image of case (3) this lesion exhibits T1W iso- to- hypointensity, with prominent peripheral contrast enhancement. **d** Representative T1W MR image of case (4) this lesion exhibits T1W hypointensity, with prominent peripheral contrast enhancement. **e** Representative T1W MR image of case (5) this lesion exhibits T1W hyperintensity, with subtle contrast enhancement and central heterogeneous signal. **f** Representative T1W MR images of case (6) the sellar lesion demonstrates heterogeneous signal centrally, with a peripheral rim of contrast enhancement. The satellite lesion adjacent to the sella on the left-hand side exhibits similar signal characteristics

The most common imaging features, present in 100% (n = 6) of patients were: sellar expansion; a cystic nature to the tumour, some degree of contrast enhancment, and localisation of the tumour epicentre in quadrant 1 (suprasellar, preinfundibular region). Five patients (83%) had the presence of stalk thickening, and some degree of lesion hyperintensity on T1-weighted (T1W) images (Fig. 2). 67% of patients (n = 4) had optic nerve compression pre-operatively. Xanthogranuloma was not listed in any of the patients’ differential diagnosis based on pre-operative imaging features (Table 2). The most common radiological differential diagnosis was craniopharyngioma, reported as the most likely lesion in 50% (n = 3) of patients. The mean maximum diameter of tumours was 20 mm (median 18 mm, range 13–29 mm). There was no calcification or cavernous sinus invasion in any cases. Pre-operative imaging features are further outlined in Table 2 and defining features outlined in Fig. 2. All cases are demonstrated in Fig. 3.

**Table 2:** Pre-operative imaging features of pituitary xanthogranulomas

	Case 1	Case 2	Case 3	Case 4	Case 5	Case 6
Cystic	+	+	+	+	+	+
Rim enhancment	+	–	–	+	–	+
Central enhancement	–	+	+	–	+	+
Stalk thickening	+	–	+	+	+	+
Stalk enhancement	+	–	–	–	–	–
Maximum diameter (mm)	13	14	18	18	26	29
T1W MR signal	Hyperintense	Hyperintense	Hypointense	Hypointense	Hyperintense-mixed	Hyperintense-mixed
Cavernous sinus invasion	–	–	–	–	–	–
Optic nerve compression	–	–	+	+	+	+
Sella expanded	+	+	+	+	+	+
Calcification	–	–	–	–	–	–
Quadrant	1 and 2	1 and 2	1	1 and 2	1	1
Differential diagnosis (pre-op)	Lymphocytic hypophysitis/abscess	Craniopharyngioma or pituitary apoplexy	Cystic pituitary adenoma/ RCC	Apoplexy/non-functioning adenoma	Craniopharyngioma	Craniopharyngioma

– indicates absence, + indicates presence

### Post-operative hormone status

Prolactin levels completely normalised in all patients after surgery. Five patients (83%) required steroid replacement post-operatively. One of these patients had a normal steroid axis at 4 months postoperatively and hydrocortisone replacement was thus ceased, leaving 67%, (4/6) of patients requiring permanent steroid replacement (Table 3). Similarly, four patients (67%) also suffered with persistent secondary hypothyroidism. One-third (2/6) of patients had growth hormone deficiency, one of which developed post-operatively. Two patients developed diabetes insipidus (DI) post-operatively, necessitating desmopressin therapy. One of these cases was transient and did not require long-term hormonal treatment. The other case of persistent DI is attributed to pituitary stalk transection during surgery, a decision made to facilitate complete removal of the tumour. One patient did not require any hormonal supplementation at the end of the analysed follow-up period (median 33.5 months; Table 3). The overall rates of persistent anterior hypopituitarism are 67%, and permanent DI occurred in 1 patient (16%). Amongst cases 3, 5 and 6, with tumour epicentres located in quadrant 1, (Table 2) only one patient had a normal endocrine axis post-operatively (case 6). Additionally, tumour epicentre did not correlate with post-operative posterior pituitary function.

**Table 3:** Post-operative endocrine function after endoscopic transsphenoidal resection of pituitary xanthogranuloma

	Pituitary function post op					
	Corticotrope axis	Thyrotrope axis	Gonadotrope axis	Somatotrope axis	ADH axis	Prolactin
Case 1	Transient deficiency (normalised 4 months post-operatively)	–	Normal	Normal	Normal	Normal
Case 2	–	–	–	–	Normal	Normal
Case 3	–	Normal	–	Normal	Normal	Normal
Case 4	–	–	–	Normal	–	Normal
Case 5	–	–	–	–	Normal	Normal
Case 6	Normal	Normal	Normal	Normal	Transient DI → Normal	Normal

– indicates axis deficiency

### Histology

The most common histological features identified were: multinucleated giant cells, CD68 positivity, and squamoid epithelium. These features were present in 67% (n = 4) of patients (Table 4). Haemosiderin and macrophages were found in 50% of patients. Granulomas, cholesterol clefts, granulation tissue, histiocytes, T and B lymphocytes, necrosis and plasma cells were found in 33% (n = 2) of patients.

**Table 4:** Histological features of tissue from 6 cases of pituitary xanthogranuloma treated by endoscopic endonasal resection

	Case 1	Case 2	Case 3	Case 4	Case 5	Case 6
Multinucleated giant cells	–	+	–	+	+	+
CD 68	+	–	+	+	+	–
Haemosiderin	–	+	–	–	+	+
Granuloma	–	+	–	+	–	–
Cholesterol clefts	–	–	–	–	+	+
Granulation tissue	–	+	–	–	–	+
Squamoid epithelium	–	+	+	–	+	+
Histiocytes	–	–	–	+	+	–
Foamy macrophages	–	–	+	+	+	–
T and B lymphocytes	–	–	–	+	+	–
Eosinophils	–	–	–	–	–	–
Necrosis	+	–	–	+	–	–
Plasma cells	–	–	+	+	–	–

+ indicates presence, – indicates absence

### Visual status pre- and post-operatively

Sixty-seven percent (n = 4) of patients presented with visual defects, most commonly a bitemporal hemianopia (n = 3). One patient (17%) patient presented with complete loss of vision in one eye (Table 5). However, post-operative visual fields and visual acuity were recorded as normal in all six patients, regardless as to whether tumour epicentre was located in quadrant 1 or quadrants 1 and 2.

**Table 5:** Comparison of pre- and post-operative visual function in cases of pituitary xanthogranuloma

	Visual function	
	Pre-op	Post op
Case 1	Normal	Normal
Case 2	Normal	Normal
Case 3	Incomplete bitemporal hemianopia	Normal
Case 4	Incomplete bitemporal hemianopia	Normal
Case 5	Incomplete bitemporal hemianopia	Normal
Case 6	Left eye—hand movements Right eye—normal	Normal

### Extent of resection and recurrence

Complete GTR was achieved in 100% (n = 6) of patients. One patient required two surgeries to achieve this level of tumour clearance (Case 6); following the first surgery, a transcranial approach for a laterally based cyst, residual tumour was identified on routine 6-month follow-up imaging. This residual tumour mass was subsequently resected completely via an endonasal endoscopic approach.

There was no post-operative CSF leak in any patients in the cohort. The pituitary stalk was preserved in 83% (n = 5) of patients. In the other case, the tumour mass was adherent to the stalk itself. The decision was made that the benefit of achieving GTR outweighed the disadvantage of developing endocrine deficits, as the diagnosis was thought to be craniopharyngioma, (Case 4; Table 3).

No tumour recurrence has occurred to date in any patient after complete resection at a median follow-up of 33.5 months (range 6–90 months). Additionally, no patients have developed any other systemic evidence of inflammatory or granulomatous disease elsewhere.

## Discussion

Intracranial xanthogranulomas arise most commonly in the choroid plexus at the trigone of the lateral ventricle; xanthogranulomas of the sellar region are much rarer [3]. The entity was first identified in 1988 [5], and was placed on the spectrum of cystic pathology occurring in the sellar region, incorporating CPs, RCCs, colloid cysts, cystic adenomas, and dermoid cysts [6]. Paulus et al. [7] described xanthogranuloma of the sellar region as a distinct entity in 1999, identifying 37 cases wherein resected pituitary lesions consisted predominantly of xanthogranulomatous components once examined histologically. These lesions may be an individual disease entity, [7] or represent the outcome after repetitive inflammation and haemorrhage into a previously typical RCC or CP.

### Demographics

Previous literature has reported the prevalence of sellar and parasellar xanthogranulomas in pituitary tumour patients the western hemisphere to be in the region of 0.6% [2]. The prevalence of xanthogranulomas in our study was 2% (6/295), in keeping with another recent series which reported just under 3% of their pituitary tumour database comprised of xanthogranulomatous lesions (6/223) [3, 8]. Whilst it remains rare, as awareness of this disease entity has developed it appears to be more commonly identified on histology within the cohort of patients with cystic sellar and parasellar tumours.

Four patients in the studied cohort were female (67%). Larger case series have also identified a slight female preponderance, with one recent study identifying 16 (59%) females and 11 (41%) males within their 27-patient cohort [4]. The mean age of patients in this study was 29, in keeping with the largest case series of xanthogranulomatous pituitary lesions to date, which stated a mean age 27 [7]. Others have reported mean ages of their pituitary xanthogranuloma patients as slightly older (43–46 years) [2, 9]. This discrepancy likely reflects the low numbers of cases in each cohort. However, a trend towards younger patients is noted, with only one case reported in a patient over the age of 70 [7].

The most common presenting features in our cohort were visual deterioration and clinical endocrine dysfunction, at least one of which was present in 67% (n = 4) of cases. The most common visual deficit was a bitemporal hemianopia (n = 3). Three patients (50%) also presented with headaches. These clinical manifestations are similar to those reported in other series, with endocrine dysfunction, (e.g. diabetes insipidus, hypoadrenalism) often reported as the most common presenting feature [3, 10, 11].

All patients with visual field deficits in our study regained normal vision post-operatively. Concordantly, almost all patients discussed in the literature are reported to have normal or near-baseline levels of vision or post-operatively. Conversely, endocrinological deficits are rarely reported to improve after surgery [4, 9].

### Endocrine effects

Four (67%) of our patient cohort presented with biochemical hyperprolactinaemia. Pre-operative trials of dopamine agonists had no effect on tumour size or serum prolactin and hyperprolactinaemia resolved in all cases after surgery. In total, one patient was free of any hormonal supplementation (case 6), and one demonstrated improvement in endocrine function, (case 1) at their most recent follow-up (Table 3).

These endocrine outcomes are slightly more promising than those previously reported, as in the majority of published cases endocrine function rarely improves after xanthogranuloma resection and most patients remain on long-term hormonal supplementation. For instance, Amano et al. reported that in their cohort of seven pituitary xanthogranuloma patients, 6 cases (86%) presented with pituitary hormonal dysfunction, and this persisted in all six patients after surgery (3 total resection, 3 sub-total resection) [9].

In a larger analysis of parasellar xanthogranuloma patients, the most common presenting symptom was an endocrinological deficit (74%; n = 20/27), usually either isolated DI or panhypopituitarism. All 20 cases had persistence of their endocrine deficiency post-operatively, with a total of 21 patients (77%) requiring hormonal treatment despite adequate removal of the pituitary xanthogranuloma [4].

The risk of permanent pituitary dysfunction with sellar xanthogranulomas may reflect the time interval between symptom onset and surgery, and/or the repetitive inflammatory and haemorrhagic nature of the lesions causing ongoing damage to normal pituitary parenchyma. Therefore, the ability to more confidently identify suspected cases of xanthogranulomatous pituitary masses is desirable, as this could justify expediting surgical intervention, potentially permitting more favourable endocrine outcomes [2, 9]. It is also possible that meticulous dissection, focus on optic apparatus decompression, and preservation of the pituitary stalk when feasible may have contributed to the favourable endocrine outcomes achieved in a third of patients in our series. In our single case of permanent DI, a decision was taken to sacrifice the pituitary stalk in the aim of curative resection of a craniopharyngioma; this was probably not necessary given the pathology of xanthogranuloma, and could have been avoided if the diagnosis was suspected pre-operatively. Similarly, the high rate of anterior hypopituitarism following surgery compared to surgery for pituitary adenoma, highlights the need for clinical suspicion of the diagnosis pre-operatively to aid patient counselling.

### Imaging appearances

No typical radiological signs are thought to highlight xanthogranuloma as the most likely possibility in the differential diagnosis of sellar lesions [6]. This is reflected in the fact that xanthogranuloma was not listed in the radiological differential diagnosis for any patients in our cohort. The most common radiological differential diagnosis was craniopharyngioma, which was documented as the most likely diagnosis for 3 patients, (50%) prior to their surgeries in the present study.

It has been speculated that cholesterol components within these lesions should be identifiable from their T1W hyper-intensity and T2W hypo-intensity on pituitary MR scans, [6, 12]. However, fluid components within the cystic lesions can appear hyperintense on T2W images, and the development of more profound fibrosis (granulation) and haemorrhage can appear as both T1W and T2W hypointensities [6]. These mixed signal intensities reflect the complex histologic components that make up granulomatous inflammatory lesions. Eighty-three percent (5/6) of cases in our cohort demonstrated a degree of T1W hyperintensity of their pituitary lesions. This is consistent with imaging descriptions in other case reports [4, 10, 13], and may represent the cystic content and cholesterol clefts before any haemorrhage or degeneration of lesion has occurred. However, once a degree of necrosis or haemorrhage occurs, the lesions may then demonstrate heterogeneous signals on both T1W and T2W images, making their differentiation from other cystic lesions such as RCC and CPs extremely challenging.

All six patients in this case series demonstrated some degree of contrast enhancement of their parasellar lesions; three cases (50%) demonstrated peripheral rim enhancement of their pituitary lesion on post-contrast T1W scans. Amano et al. [9] specified that 71% (5/7) of sellar xanthogranulomas in their series also demonstrated T1W peripheral rim enhancement. The other three cases in our cohort did also display contract enhancement, but in a more homogenous manner. RCCs do not typically enhance on MRI scans post-contrast, and calcification is rarely identified. CPs typically demonstrate heterogeneous contrast enhancement, and frequently do have calcification identifiable within the cystic lesion, particularly in the adamantinomatous sub-type. There was no calcification seen in any of the pre-operative MRI or CT head scans in our cohort, and thus far only 7 reported cases of pituitary xanthogranulomas have demonstrated calcification on imaging [3, 10].

Furthermore, no cases in our cohort demonstrated cavernous sinus involvement, which has been reported for both RCCs and CPs [14]. Cavernous sinus involvement remains as yet unreported in pituitary xanthogranulomas [4, 9, 10, 13]. The absence of calcification and cavernous sinus invasion, in the setting of peripheral or global contrast enhancement, may therefore constitute a constellation of imaging features that necessitate consideration of xanthogranuloma in the pre-operative differential diagnosis.

Table 6 provides a summary of typical features of CPs, RCCs and pituitary xanthogranulomas on conventional MR imaging. However, these features are not universal to any of the lesions, and therefore there are currently no specific radiological criteria which can be used differentiate xanthogranuloma from other cystic sellar lesions with complete confidence.

**Table 6:** Summary of typical features of CP, RCC and pituitary xanthogranulomas on MRI

	RCC	CP	Xanthogranuloma
T1W	Hyperintense	Heterogeneous	Hyper- and/or hypointense
T2W	Hypointense	Heterogeneous	Hyper- or hypointense
Contrast enhancement	–	Diffuse or peripheral enhancement	Peripheral rim or homogeneous enhancement
Calcification (on CT scan)	–	+	Rare
Tumour epicentre	Usually sellar	Usually suprasellar	Suprasellar
Cavernous sinus invasion	May be present	May be present	Unreported

+ indicates typically present; – indicates typically absent

All lesions analysed in this study had tumour epicentres which were universally suprasellar, (Quadrants 1–2) which also appears to be a consistent finding in other case series [3]. This contrasts to cystic pituitary adenomas and RCCs, which typically have an epicentre located in sellar quadrants, (quadrants 3 or 4). This supports the notion that pituitary xanthogranulomas may hold their origin in the hypothalamoinfundibular region [2, 9]. A primarily sellar tumour epicentre may therefore suggest cystic adenoma or RCC as more likely diagnoses when assessing cystic pituitary lesions.

Non-infectious, xanthogranulomatous inflammation of the frontal lobe have been shown to demonstrate restriction of diffusion on diffusion-weighted MR scans [15]. The features of CPs and RCCs with diffusion weighted imaging (DWI) and MR spectroscopy have been assessed, [11, 12] and therefore specific analysis of DWI appearances in patients with sellar xanthogranuloma is warranted. If clear differences in diffusion restriction between these lesions are identified, this modality has the potential to aid clinicians in differentiating pituitary xanthogranulomas from other cystic sellar lesions prior to surgery.

### Histology

Similarities in histological appearances and the consistent presence of chronic inflammatory cells and debris in sellar xanthogranulomas has fuelled the theory that these lesions are on the same spectrum as RCC’s and CPs, potentially representing a secondary reaction caused by repeated inflammation and haemorrhage [16]. RCCs consists of simple columnar/cuboidal epithelium, whereas CPs are divided into histological sub-types: adamantinomatous, (stratified squamous epithelium with keratin nodules +/− calcification) or papillary (squamous epithelium).

At least two or more of the characteristic features of xanthogranulomas, such as cholesterol clefts, foamy macrophages, granulomas, necrosis, and/or haemosiderin deposition [4, 7, 9] were identified in all patients within the study group. In addition, squamous metaplasia was present in 67% (4/6) of patients. Le et al. [17] reported that 39%; (11/28) of RCCs displayed squamous metaplasia, and that xanthogranulomatous components were identified in 13/28 confirmed RCCs (47%). Conversely, xanthogranuloma-like features were highlighted in comparatively fewer (n = 5/25; 20%) CPs. Amano et al. identified components of RCCs (cuboidal/columnar epithelium, squamous metaplasia) within 86% (n = 6/7) of resected pituitary lesions which otherwise composed primarily of xanthogranlomatous material [9]. The authors proposed that pituitary xanthogranulomas may represent a final inflammatory state, resulting from secondary reactions caused by repeated haemorrhage, inflammation and degeneration of RCCs.

However, identification of residual RCC/CP components within pituitary xanthogranulomas is not reported consistently in histological studies across the literature [1, 6, 9, 13]. Furthermore, xanthogranuloma histology features are similar across multiple sites both intracranially and elsewhere in the body, and thus they may represent a unique disease entity [6, 18].

However, this may be a function of heterogeneity within the tissues sent for analysis from pituitary lesions, and thus it remains possible that xanthogranulomas represent one condition a spectrum of cystic sellar and parasellar lesions, or a stereotyped response to repeated inflammation and haemorrhage within a biological structure [18]. Future studies of cystic pituitary lesions must ensure that sufficient quantities of tissue are sent for histological analysis, to minimise the risk of sampling bias when analysing these microscopically heterogeneous lesions.

### Treatment

Whilst radiotherapy for intracranial xanthogranulomas at other sites has been utilised, its use in sellar lesions is yet to be evaluated, partly due to the diagnostic uncertainty inherent when planning treatment of cystic lesions in this region. GTR is considered gold standard treatment for these lesions. The endoscopic endonasal approach is generally favoured over transcranial approaches when feasible [6]. In all patients in our cohort, visual function was normal post-operatively, in contrast to endocrine outcomes, which improved in 2 patients after long-term follow-up. Recurrence or re-growth was not reported in any case during the present study’s follow-up period, and recurrence is rarely noted in other case reports, regardless whether total or subtotal resection was performed at the initial operation [1, 4]. Given these factors, tumour mass reduction to decompress the optic chiasm, with sparing of the pituitary stalk if not involved in the lesion, may be regarded as the appropriate surgical target when planning resection of a suspected xanthogranuloma [9].

However, GTR is advocated for RCCs and CPs. Therefore, until definitive clinical or radiological diagnostic features to identify pituitary xanthogranulomas prior to surgery are elucidated, GTR may need to continue to be the surgical goal for cystic sellar/parasellar lesions. As the long-term prognosis of xanthogranulomatous pituitary lesions after surgical removal is yet to be evaluated, close clinical and radiologic follow-up is indicated [2, 16, 18].

## Conclusion

The prevalence of pituitary xanthogranulomas found in our cohort is slightly higher than previously reported in the literature. These patients tend to be young at presentation, and are likely to present with endocrinological disturbances and/or visual deficits. The prognosis for visual function is excellent, with most patients having normal or improved vision following surgery. The rate of post-operative anterior hypopituitarism is high, but permanent DI is much less frequent. Preservation of the pituitary stalk may contribute to more favourable endocrine outcomes, particularly given the low recurrence rates reported even after subtotal resections of pituitary xanthogranulomas [9, 10].

Imaging features such as a degree of T1W hyper-intensity alongside either peripheral or diffuse contrast enhancement have been identified in numerous patients with pituitary xanthogranulomas. If these features are identified alongside a primariy suprasellar tumour epicentre, an absence of cavernous sinus invasion, and no identifiable calcification, the possibility of sellar/parasellar xanthogranuloma must be considered. Appreciation of this rare but important condition within the differential diagnosis can impact upon patient counselling and peri-operative planning from both surgical and endocrinological points of view. Characterisation of how these lesions appear on advanced imaging modalities, such as diffusion weighted imaging, warrants exploration.
